# An Unfortunate Incident

**Published:** 1896-02

**Authors:** 


### AN UNFORTUNATE INCIDENT.

THE implanting of home customs on foreign soil or in foreign institutions is too fraught with danger to be given encouragement any longer. Often a day, in these swiftly changing times, strains the relations for a time between the most friendly countries. The incident at the Toronto Veterinary College on Thanksgiving Day last must be abhorred and regretted by all, and the results must be disastrous to that school in lessening its great quota of students annually recruited from the United States. If the custom has annually prevailed for a period of years of unfurling the American flag in the college class-room on that auspicious day, there can be little said in extenuation of the insult given it by those of another country. A custom that cannot safely be encouraged should, therefore, be withheld.

---

THE veterinarians of the District of Columbia in their desire to obtain at the hands of Congress a bill regulating the practice and establishment of a Board of Examiners, should not imperil the success of the bill by demanding that said appointments shall be restricted to members of the local association, when said body does not include all the good men of their district.

The importation of uninspected English bacon to our ports for consumption by fastidious customers is one of the anomalies of the day, while every pound for export has to undergo thorough inspection.

Veterinarians of Illinois are considering the necessity of greater safeguards for the people from the imposition of ignorant pretenders who rob the public under the guise of veterinarians. The establishment of a State Board of Veterinary Examiners is being urged, and the necessity of registration is now realized.

